# Emerging viral threats in Gabon: health capacities and response to the risk of emerging zoonotic diseases in Central Africa

**DOI:** 10.3134/ehtj.10.163

**Published:** 2010-06-03

**Authors:** M Bourgarel, N Wauquier, J-P Gonzalez

**Affiliations:** 1 Centre de Coopération Internationale en Recherche Agronomique pour le Développement (CIRAD), UPR AGIRs, Campus International de Baillarguet, Montpellier cedex 5, France; 2 Centre International de Recherches Médicales de Franceville (CIRMF), Unité de Recherche Ecologie de la Santé, Franceville, Gabon; 3 Centre International de Recherches Médicales de Franceville (CIRMF), Unité des Maladies Virales émergentes, Franceville, Gabon

## Abstract

Emerging infectious diseases (EID) are currently the major threat to public health worldwide and most EID events have involved zoonotic infectious agents. Central Africa in general and Gabon in particular are privileged areas for the emergence of zoonotic EIDs. Indeed, human incursions in Gabonese forests for exploitation purposes lead to intensified contacts between humans and wildlife thus generating an increased risk of emergence of zoonotic diseases. In Gabon, 51 endemic or potential endemic viral infectious diseases have been reported. Among them, 22 are of zoonotic origin and involve 12 families of viruses. The most notorious are dengue, yellow fever, ebola, marburg, Rift Valley fever and chikungunya viruses. Potential EID due to wildlife in Gabon are thereby plentiful and need to be inventoried. The Gabonese Public Health system covers geographically most of the country allowing a good access to sanitary information and efficient monitoring of emerging diseases. However, access to treatment and prevention is better in urban areas where medical structures are more developed and financial means are concentrated even though the population is equally distributed between urban and rural areas. In spite of this, Gabon could be a good field for investigating the emergence or re-emergence of zoonotic EID. Indeed Gabonese health research structures such as CIRMF, advantageously located, offer high quality researchers and facilities that study pathogens and wildlife ecology, aiming toward a better understanding of the contact and transmission mechanisms of new pathogens from wildlife to human, the emergence of zoonotic EID and the breaking of species barriers by pathogens.

## Introduction

Despite intensive research and considerable effort from public health agencies to prevent or eradicate infectious diseases, emerging infectious diseases (EID) are currently the major threat to public health worldwide.1 Indeed, many new infectious agents, characterized by a high pathogenic potential, have been recently identified. Furthermore, some well known pathogens have been expanding their territories, causing increasing concerns in the recent decades due to changing epidemiological patterns.2 Most of these EID events have involved zoonotic infectious agents: more than 60% of EID affecting humans have a zoonotic origin^3^,^4^ and B75% of the diseases that have emerged over the past two decades have wildlife sources.5 Therefore, zoonotic EID represent a major and increasing threat to global health.1,3,6 Zoonoses refer to infectious diseases that are susceptible to be transmitted from animals to humans and are responsible worldwide for a great deal of pain, morbidity and even human fatalities. Two categories of zoonotic diseases have been described:4 (1) diseases for which transmission events to humans are rare but once occurred, horizontal transmission from human-to-human maintains a more or less sustainable infectious cycle (for example: ebola virus, from naturally infected chiropteran to human epidemics); (2) diseases for which direct or vector-mediated animal-tohuman transmission remains the common source of human infection (for example: Rift Valley fever virus (RVFV), mosquito-transmitted from infected domestic ungulates).

Emerging and re-emerging zoonoses include recently identified infectious diseases, diseases that have recently evolved from a subclinical state to a clinical syndrome, and previously known diseases that have recently displayed an increase in incidence or that have spread to new regions, hosts or vectors.4 However, a disease may not be recognized as zoonotic at the first outset. The disease can spread undetected for a period of time depending on the incubation period (weeks to years), the epidemiological pattern of a subclinical disease to a clinical picture (the emergence of the symptoms depending on host or pathogen factors) or, if the number of cases, in both human and animal populations, is too small and undetectable during the initial stages of transmission to suspect a link between the two events.^7^

Zoonotic EID outbreaks result from a now classically accepted phenomenon of concurrency of fundamentals and territories of emergence. Fundamentals include factors related to the host, the vector (if any), the pathogen, and favorable environmental factors (climate) although territories at risk are the product of human activity and high-risk behavior among the human population. For instance, territories (that is: city, district) with an inefficient disease detection system or a failure to control vectors and other carriers of diseases as well as man-made environmental changes (breakdown of the water system, deforestationy) will force an increase in contact between the human population and wildlife.^2^, ^8^

Tropical forests form the ecosystem harboring the highest species richness of all terrestrial ecosystems and shelter almost 50% of the total global biodiversity.^9^, ^10^ This includes wildlife, flora, multi-cellular organisms as well as an immense diversity of pathogens including bacteria, parasites and viruses. Actually, there is a latitudinal spatial gradient of pathogenic species richness increasing towards the Equator. 11 After the Amazonian Basin, the Congo basin in Central Africa has the world's second largest contiguous block of tropical rainforest, which encompasses many areas that remain largely undisturbed, due in large part to low human population densities and the remoteness of interior rainforests. 12 As wildlife host species richness is a good predictor for the emergence of zoonotic EIDs with a wildlife origin,^3^ Central Africa in general, and Gabon in particular are privileged areas for the emergence of zoonotic EIDs.

### The potentialities of emerging zoonotic diseases from wild to domestic environments

Latest reviews on EID show that nearly 75% of zoonotic EID have a wildlife origin.^3^, ^5^, ^13^–^15^ In fact, the number of EID events caused by pathogens coming from wildlife has increased during the past six decades.^3^ The majority of pathogens recorded were of viral origin.^16^ Therefore viral zoonoses of wildlife origin represent the most significant and growing threat to global health among all EIDs.^3^, ^13^

As anthropogenic activities have been identified as the cause of a significant majority of outbreaks,^16^, ^17^ it is essential to fully understand the mechanisms driving contacts between wildlife and the human population as well as species-jumping infections to set up public health information campaigns. On the contrary, efforts to conserve areas rich in wildlife diversity (13 National Parks were created in 2002 in Gabon) by reducing anthropogenic activity may have an added value in reducing the likelihood of future zoonotic disease emergence in these areas.^3^ EIDs in free-living wild animals can be classified into three major groups on the basis of key epizootiological criteria:^18^ (i) EIDs associated with ‘spill-over’ from domestic animals to wildlife populations living in proximity; (ii) EIDs related directly to human intervention, via host or parasite translocations; and (iii) EIDs with no overt human or domestic animal involvement. These phenomena have two major biological implications: first, many wildlife species are reservoirs of pathogens that threaten domestic animal and human health; second, wildlife EIDs pose a substantial threat to the conservation of global biodiversity, with for example the disappearance of the most great ape populations in protected areas in Central Africa after the 2002–2003 ebola virus outbreaks.^19^–^22^

### Emerging zoonotic diseases in Gabon

In Gabon, rainforests cover ∼80% of the territory.^23^ These forests are known for their rich biodiversity of animal and plant species.^23^ The human population, estimated at 1.5 million,^24^ lives more in cities (54%) than in rural areas.^25^ Gabon's economy has relied in the past mainly on petroleum exports, forest exploitation, and mining activities. Forest habitats are now exploited by logging and mining companies, tourism and hunting activities, which produce about 17,500 metric tons per year of game meat. Altogether these human incursions in Gabonese forests for exploitation purposes lead to intensified contacts between humans and wildlife and generate a risk of emergence of zoonotic diseases.^26^ This risk is not strictly restricted to the forest but exists countrywide as the urban demand for bush-meat in Gabon is important >5 kg/per/year^27^. Indeed, every Gabonese city has traditional and local food markets where fresh and smoked bush-meat coming from all around the country are available. Potential EIDs due to wildlife in Gabon are thereby plentiful and need to be inventoried.

#### Known emerging viral diseases in Gabon

At least 51 endemic or potential endemic viral infectious diseases have been reported in Gabon^28^ (Table 1). Among them, 22 are of zoonotic origin and involve 12 families of viruses. The most represented are Flaviviridae (dengue virus, yellow fever virus (YFV), zika fever virus), Poxviridae (monkeypox virus (MPXV)), Filoviridae (ebola and Marburg Viruses), Arenaviridae (lassa fever virus), Bunyaviridae (RVFV) and Togaviridae (chikungunya virus).

**Table 1:** Viral infections described, or potentially present, in Gabon

	*Diseases*	*Virus*	*Main vector or infectious mean*	*Epidemiological state*			*Reference*
				
				*Reservoir present*	*Clinical epidemiology*	*Public health action in Gabon*	
1	Acquired immune deficiency syndrome (AIDS)	Retroviridae, lentivirus: human immunodeficiency virus	Blood, semen, transplacental	Human	1983: first case	Tri therapy, Ambulatory Treatment Centers (CTA)	69
					1994: prevalence of 0.8% (Franceville); 1.7% (Libreville) 2005: ∼60,000 cases		
2	Bunyaviridae infections (fever, headache)	Bunyaviridae, bunyavirus: orthobunyavirus	Mosquito	Rat, (bird, chipmunk, cattle, sheep, bat)^a^	Mild infection and endemic or potentially endemic	Supportive treatment	28
3	Chikungunya fever	Togaviridae, alphavirus: chikungunya virus	Mosquito *(A. aibopictus)*	Non-human primate	2006 and 2007, two outbreaks 1 7,618 cases in Libreville	Supportive treatment	33, 70
4	Common cold	Picornaviridae: rhinovirus and Coronaviridae: coronavirus	Droplet, direct contact	Human	Endemic	Supportive treatment	28
5	Conjunctivitis, respiratory infections, diarrhea	Adenoviridae: adenovirus	Droplet, free water	Human; non-human primates	Endemic	Vaccine, supportive treatment, hygiene and prevention (enteric/secretion), symptomatic therapy, drug: Cidofovir®	28
6	Cytomegalovirus infection	Herpesviridae: cytomegalovirus or human herpesvirus 5 (HHV-5)	Droplet (respiratory), urine, dairy products, tears, stool, sexual contact (rare)	Human	Endemic	Vaccine, drug: Ganciclovir	28
7	Dengue fever	Flaviviridae, flavivirus: dengue virus	Mosquito (A *aibopictus),* blood	Unknown	Outbreak (321 cases): 2007	Hospital survey, pilot project on hemorrhagic fever, neuronal and gastroenteritis (CIRMF)	34
8	Ebola hemorrhagic fever	Filoviridae, filovirus: ebola virus	Infected body secretions	Bat, infected animals	Outbreaks (227 cases) in1994, 1996, 2001 and 2002	CIRMF: WHO reference laboratory	29, 71
9	Gastrointestinal infection	Picornaviridae: coxsackievirus, ECHO virus, enterovirus, paraechovirus	Droplet, fecal-oral	Human	Endemic		28
10	Viral gastroenteritis	Reoviridae: rotavirus, Caliciviridae: calicivirus, Coronaviridae: torovirus, Astroviridae: astrovirus	Food, water	Human	Endemic or potentially endemic	Supportive therapy	
11P	Hantavirus infection	Bunyaviridae, hantavirus	Animal excreta	Field mouse, Rat (bat, bird)^3^	Endemic or potentially endemic	Supportive therapy	28
12	Hepatitis A	Picornaviridae, hepatovirus:hepatitis A virus	Fecal-oral, food, water, fly	Human and non-human primate	Endemic or potentially endemic	Supportive therapy, vaccine	28
13	Hepatitis B	Hepadnaviridae, orthohepadnavirus: hepatitis B virus	Blood, infected secretions, sexual contact	Human non-human primate	HBsAg-positive: Urban areas:12.9% Rural areas: 7.6%	Vaccine coverage (2008): 82%	28, 72
14	Hepatitis C	Flaviviridae, hepacivirus: hepatitis C virus	Blood, sexual contact, vertical transmission	Human	Seroprevalence (1997): Nationwide: 6.50% Pregnant women: 2.4% Adults (rural): 20.7%	Supportive therapy	73, 74
15	Hepatitis D	Deltaviridae, deltavirus: hepatitis D virus	Infected secretions, blood, sexual contact	Human	HBsAg-positive: Rural areas: 8.5% Pregnant women: 15.6%	Supportive therapy	75, 76
16	Hepatitis E	Hepeviridae, hepevirus: hepatitis E	Fecal-oral water, shellfish, blood (rare), meat (rare)	Human, rodent, pig	Pregnant women: 14.1%	Stool precautions; supportive therapy	75, 77
17	Hepatitis G	Flaviviridae, hepacivirus: hepatitis G virus	Blood, vertical and sexual transmission suspected	Human	Pregnant women: Franceville: 11.4% Libreville: 13.3%	Supportive therapy	28
18	Herpes B infection	Herpesviridae, Alphaherpesviridae, simplexvirus: cercopithecine herpesvirus 1	Contact or bite	Monkey	Endemic or potentially endemic	Supportive therapy	28
19	Herpes simplex encephalitis	Herpesviridae, Alphaherpesvirinae, simplexvirus: human herpesvirus	Infected secretions, including Sexual contact	Human	Endemic or potentially endemic Seroprevalence in Libreville: 66% of pregnant women	Supportive therapy	28
20	Herpes simplex infection	Herpesviridae, Alphaherpesvirinae, simplexvirus: human herpesvirus 1 and II	Infected secretions, sexual contact	Human	Seroprevalence in Libreville: 66% of pregnant women	Supportive therapy	78
21	Herpes zoster	Herpesviridae, Alpha herpesvirinae: varicella-zoster	Air, direct contact	Human	Prevalence: 18.5% of HIV-positive patients	Supportive therapy	79
22	Infectious mononucleosis	EBV Herpesviridae, gammaherpesvirina, lymphocryptovirus: human herpesvirus 4	Saliva, blood transfusion	Human	Endemic or potentially endemic	Supportive therapy	28
23	Influenza	Orthomyxoviridae, orthomyxovirus: influenza virus	Droplet	Human, ferret, pig, bird	one case reported	Respiratory precautions, a neuraminidase inhibitor and vaccines, CIRMF: N1N1 and H5N1 focal laboratory	28
24	Laryngotracheobronchitis	Paramyxoviridae, Respirovirus: parainfluenza Virus	Droplet	Human	Endemic or potentially endemic	Supportive therapy	28
25	Lassa fever	Arenaviridae, arenavirus: lassa virus	Rodent secretions, dust, food, patient secretions	Multimammate rat	Endemic or potentially endemic	Strict isolation	28
26	Lymphocytic choriomeningitis	Arenaviridae, arenavirus: lymphocytic choriomeningitis	Urine, saliva, feces, food, dust	House mouse, guinea pig, hamster, monkey	Endemic or potentially endemic	Supportive therapy	28
27	Marburg virus disease	Filoviridae, filovirus: Marburg virus	Infected secretions contact, syringe, needle	Bat, Other?	No human case reported Seroprevalence in bats: 1%	Strict isolation, supportive therapy	80
28	Measles	Paramyxoviridae, morbillivirus: measles virus	Droplet	Human		Respiratory isolation, supportive therapy, 67% vaccine coverage (2008)	28
29	Aseptic Meningitis	Picornaviridae, enteroviruses	Fecal-oral; Droplet	Human	Endemic or potentially endemic	Supportive therapy	28
30	Monkeypox	Poxviridae, orthopoxvirus: monkeypox virus	Contact	Monkey, squirrel, r odent	1991: First human cases of monkeypox reported (four siblings, two fatal)	Strict isolation, supportive therapy	81
31	Mumps	Paramyxoviridae, rubulavirus: mumps virus	Aerosol	Human	2001: 934 cases reported	Respiratory isolation, supportive therapy	28
32	Orf	Poxviridae, parapoxvirus: orf virus	Contact, infected secretions, fomite	Sheep, goat, reindeer, musk ox	Endemic or potentially endemic	Supportive therapy	28
33	Parainfluenza virus infection	Paramyxoviridae: respirovirus-human parainfluenza virus 1 and 3. rubulavirus-human parainfluenza virus 2 and 4.	Droplet	Human	Endemic or potentially endemic	Supportive therapy	28
34	Parvovirus B19 infection	Parvoviridae: erythrovirus B19	Droplet	Human	Endemic or potentially endemic	Supportive therapy	28
35	Pleurodynia	Picornaviridae: coxsackievirus	Air, fecal-oral, fomite	Human	Endemic or potentially endemic	Supportive therapy	28
36	Poliomyelitis	Picornaviridae, picornavirus: polio virus	Fecal-oral, dairy products, food, water, fly	Human	no case since 1998	Stool precautions, supportive therapy, vaccination (2008): 81% of vaccine coverage	28
37	Pseudocowpox	Poxviridae, parapoxvirus: pseudocowpox virus	Contact	Cattle	Endemic or potentially endemic	Supportive therapy	28
38	Rabies	Rhabdoviridae, lyssavirus: rabies virus	Saliva, bite, transplants, air (bat aerosol)	Dog, fox, skunk, jackal, wolf, cat, raccoon, mongoose, bat, rodent or rabbit (rarely)	1997: 12 cases of human rabies	Strict isolation, supportive therapy, vaccination	28
39	Respiratory syncytial virus infection	Paramyxoviridae: human respiratory syncytial virus	Droplet, infected secretions (hands)	Human	Endemic or potentially endemic	Vaccination	28
40	Respiratory viral infection	Paramyxoviridae: Human metapneumovirus, Coronaviridae, Coronavirus: HKU1, New Haven Parvoviridae: human bocavirus	Droplet, Infected Secretions (hands)	Human	Endemic or potentially endemic		28
41	Rift Valley fever	Bunyaviridae, phlebovirus: Rift Valley fever virus	Mosquito	Sheep, ruminant, wildlife^3^	Endemic or potentially endemic	Supportive therapy	28
42	Roseola	Herpesviridae, betaherpesvirinae, roseolovirus: herpesvirus 6	Droplet, contact	Human	Endemic or potentially endemic	Supportive therapy	28
43	Rotavirus infection	Reoviridae: rotavirus	Fecal-oral, Water	Human	Prevalence (1985): 11-30% of gastroenteritis in children	Stool precautions, supportive therapy	
44	Rubella	Togaviridae, rubivirus: rubella virus	Contact, air, transplacental	Human	2008: 55 cases	Respiratory precautions, supportive therapy	82
45	Small pox	Poxviridae, orthopoxvirus: variola virus	Contact, infected secretions, fomite	Human	Cases reported: 1963: 111 1 964: 49 1965: 1	Isolation, supportive therapy	28
46	Spondweni	Flaviviridae, flavivirus: spondweni virus	Mosquito	Unknown	Evidence for Spondweni infection has been found in Americans residing in Gabon	Supportive therapy	83
47	Varicella	Herpesviridae, Alphaherpesvirinae: human herpesvirus 3	Air, contact	Human	Endemic or potentially endemic	Respiratory isolation,acyclovir therapy	28
48	Wesselsbron	Flaviridae, flavivirus: wesselsbron virus	Mosquito	sheep, cattle	Seropositivity documented in 1975	Supportive therapy	84
49	West Nile Fever	Flaviridae, flavivirus: west Nile virus	Mosquito	Bird, horse, bat^a^, tick	Seroprevalence: 2002-2005: 3% of horses in riding stables (Libreville, Port Gentil and Moanda)	Supportive therapy	85
50	Yellow fever	Flaviridae, flavivirus: yellow fever virus	Mosquito	Human, mosquito, monkey	Makokou (Ogooue-lvindo Province): 1994: 44 cases, 18 fatal 2006: 57 cases	Supportive therapy, 70% vaccine coverage of target population (2008)	43
51	Zika	Flaviviridae, flavivirus: zika virus	Mosquito	Human, Mosquito, Monkey	1975: Seropositivity documented	Supportive therapy	84, 86

a Not confirmed.

During the past two decades, several outbreaks of these zoonotic viral diseases have been reported in Gabon. All of them had a major impact on the public health:

- Zaïre ebola virus (ZEBOV): in Gabon, ZEBOV outbreaks occurred in 1994, 1996, 1997 and 2001;29 primary human cases were generally contaminated by direct contact with dead wild animals, such as great apes (chimpanzee and gorilla), which are highly susceptible to the disease, and therefore human outbreaks were often preceded by an animal epizootic (great apes). Since the first recorded outbreak in 1976, 20 human epidemics have occurred in Central Africa^29^, ^30^ with three recent outbreaks in RDC and Uganda in 2007 and 2008.
- Chikungunya virus (CHIKV): CHIKV has recently dispersed to new regions of the world including Gabon where two outbreaks in 2006 and 2007 mainly hit the capital, Libreville.^31^, ^32^ A total of 17,618 human cases were reported.33 The outbreaks appeared concomitantly with the spread in peridomestic urban areas of *Aedes albopictus*, the mosquito known as the main vector of the most recent epidemics of CHIKV.^34^ CHIKV disease had reemerged in 2001–2003^35^ in the Indian Ocean after a 20-year gap with a new epidemiological pattern including A. *albopictus* as the main vector of epidemics and an adapted virus strain presenting an original mutation suspected to be responsible for an increase of pathogenicity.^36^
- Dengue virus (DENV): a DENV outbreak occurred in Gabon simultaneously with the CHIKV outbreak in 2007,^33^ and concurrent infections of DENV and CHIKV have been reported in towns affected by the two outbreaks.^34^ Dengue fever and the severe form of the disease, dengue hemorrhagic fever (DHF), are caused by the world's most prevalent mosquito-borne virus.^37^ DENV is carried by *Aedes aegypti* mosquito, which is strongly affected by ecological and human drivers, but also influenced by climate (temperature, humidity and solar radiation).^37^ Although DENV was known to circulate among mosquitoes within limited areas in West Africa and East Africa, dengue fever first emerged among the African population during the epidemic of Nigeria in 1964–1968,^38^ then in Senegal in 1980^39^ and Burkina Faso and Kenya in 1982.^40^, ^41^ Since then epidemic manifestations were recorded in East Africa (Mozambique, Sudan, Djibouti, Somalia, Eritrea), in Senegal and more recently in Gabon.^34^,^42^ It seems that dengue fever is on the edge of emergence in Africa with the potential appearance of the devastating DHF that is yet to be observed on the continent.
- Yellow Fever Virus (YFV): Gabon is officially designated as an infected country. A YFV outbreak occurred in 1994 in Ogooue-Ivindo Province, North East of Gabon with 44 cases reported.^43^ More recently, in 2009, Cameroon reported a laboratory-confirmed case of yellow fever (YF).^44^ YF has become an important public health issue because of its case-fatality rate of 50% and the estimated 200,000 cases and 30,000 deaths that occur each year worldwide. Also, despite the efficiency of the YF vaccine and its inclusion in the national vaccination program, human populations situated in remote areas have a limited access to the public health system.

#### Potential emerging zoonotic diseases in Gabon

Based on serological evidence, several pathogens identified among wild or domestic animals, are suspected to infect the human populations of Gabon and therefore represent a potential threat to public health. Among them are the follows:

- Foamy virus: simian foamy virus (SFV), a retrovirus in the Spumaretrovirinae subfamily, is widely prevalent in wild-caught and captive-born non human primates.^45^, ^46^ Contamination between non-human primates and humans can occur via contact with infectious body fluids, through biting,^45^, ^47^ or when manipulating fresh bush-meat.^48^, ^49^ However, the potential for SFV to become a human disease and to spread among human populations after cross-species transmission is not yet fully understood.^50^
- Human monkeypox: Human monkeypox, caused by the MPXV, a member of the genus Orthopoxvirus, is clinically almost identical to ordinary smallpox.^51^ Humans become infected through direct contact with infected wild animals. It seems that monkeys are also incidental hosts as the reservoir species of MPXV remain unknown (most likely one or several rodents living in secondary forests of Central Africa).^51^ Epidemiological surveys recorded 47 cases of human monkeypox (7 lethal) in Central Africa (RDC, Gabon, Congo, CAR, Cameroun, Ivory Cost, Liberia, Sierra Leone and Nigeria)^52^ with possible secondary transmission in the human population. Since 1980, the large majority of cases to be reported from the DRC mainly concern children.^51^ In 72% of those cases, an animal source of infection was suspected whereas secondary transmission from human source was presumed for the remaining cases. The longest documented chain of infection did not exceed four generations of person-to person transmission. There is only little probability of a large epidemic spread of MXPV.^53^
- Rift valley fever virus (RVFV): Rift Valley fever is an African disease that affects both livestock and humans. RVF outbreaks are associated with persistent heavy rainfall, sustained flooding and appearance of large numbers of mosquitoes, the main vector. Localized heavy rainfall is seldom sufficient to create conditions for an outbreak;^54^, ^55^ Rift Valley fever is a good example of a disease that is well coupled with climatic anomalies; and Gabon is one of the African countries known to have some evidence of RVFV circulation as antibodies of the disease has been found in humans and livestock.^55^

### Impact of a changing environment

Climatic variations and extreme weather events have a profound impact on infectious diseases:^56^ for example, the emergence of vector-borne diseases is highly sensitive to changes in environmental conditions (rainfall, temperature, severe weather events).^56^ Indeed arthropod vectors (that is mosquito, ticks…) are devoid of thermostatic mechanisms, hence reproduction and survival rates are strongly affected by fluctuations in temperature.^37^ A rise in arthropodborne EID events due to climate anomalies has been observed during the 1990's.^3^ Average global temperatures are predicted to be 1.0–3.51^?^C by 2100.^57^ Further epidemic events of vector-borne diseases due to climate change are, therefore, to be expected.

Concerning other zoonotic infectious diseases like ebola, marburg or RVF, too little information is available concerning their ecology to be able to assess the impact of climate changes on the potential emergence or re-emergence of these diseases. However some preliminary reports show a strong association between wet environment (rainfall and hydrographic conditions directly dependent on climate and climate change) and the recent reemergence of ebola fever in Gabon and RDC.^58^ Also El Niño/Southern oscillation (ENSO) is the strongest naturally occurring source of climate variability around the globe,^59^ and more than 75% of RVF outbreaks between 1950 and 1988 occurred during warm ENSO event periods.^54^ Moreover, RVF epidemics between1950 and 1998 have coincided with unusually high rainfall in East Africa.^60^

### Epidemic prevention and control

#### Existing structures in Gabon (data cited below was updated in 2007, ministry of public health, Gabon^61^)

Gabon has one of the highest expenditure on health per capita in Africa. In 2006, Gabon's total health care expenses were comparable to eastern European countries and most southern American countries:^62^ between 300 and 1000 US$ per capita.

The Gabonese Public Health System comprises five different health sectors: (1) the civilian public sector under the public Health and Hygiene Ministry, (2) the public military sector under the Ministry of Defense, (3) the National Health Social Security Funding and private insurances, (4) the private health sector and (5) the traditional health sector.^61^

This public Health system geographically covers most of the country allowing a good access to sanitary information and efficient monitoring of emerging diseases. However, access to treatment and prevention is much better in urban areas where medical structures are more developed, more organized with higher level of technicality and material^61^ while the population is equally distributed between urban and rural areas (Table 2). A total of 60% of human and equipment resources from the Gabonese government are allocated to the main cities. In fact, all hospitals and clinics are found in Libreville, Port Gentil, Franceville and the surrounding areas, whereas in the distant rural areas, the numerous small health structures like Mother and Child Health Centers, Health Medical Centers, and Primary Care Health Center (Table 2) are often old with limited basic equipment and drugs.^61^ Also the medical staff is more concentrated in urban areas (Table 3). Indeed, more than 70% of the doctors and midwives are assigned to urban areas as well as 58% of the druggists. Only public nurses are equally distributed between rural and urban areas.

**Table 2:** Health structures in Gabon according to sectors, areas and population

*Health sector*	*General Referring hospital*	*Regional hospital*	*Clinic and polyclinic*	*Center of epidemiological survey*	*Mother and child health center*	*Health Medical center*	*Health center*	*Primary care health center*	*Primary care home*	*Nursing center*	*Medical diagnostic laboratory services*	*Health care facilities*	*Pharmacy*	*Population (x 1000)*
*Gabon*														
Public	1	11	0	10	11	57	32	472	97	54	0	0	0	0
Military	1	0	0	0	4	0	0	0	0	22	1	0	0	0
NSS^a^	0	3	1	0	0	9	0	0	0	0	0	0	0	0
Private	0	3	35	0	0	0	0	0	0	43	10	107	155	0
Total	**2**	**17**	**36**	**10**	**15**	**66**	**32**	**472**	**97**	**119**	**11**	**107**	**155**	**1518**
Ratio^b^	759	89	42	151	101	22	47	3	16	13	138	14	10	
*Urban areas*														
	2	5	25	3	2	2	16	46	3	37	7	73	51	755
*Ratio*^b^	378	151	30	252	378	378	47	16	252	20	108	10	15	
*Rural areas*														
	0	6	11	7	13	64	16	426	94	82	4	34	104	762
*Ratio*^b^	—	127	69	109	59	11	48	2	8	9	191	22	7	

Note: urban areas include Libreville, Port Gentil, Franceville and surroundings.^61^

a National Health Social security and private insurances.

b Inhabitant_1000/number of structures.

**Table 3:** Public health medical staff in Gabon according to specific environmental areas

	*Urban area*^*a*^	*Rural area*^*a*^	*Total*
Medical doctor	299 (75)	100 (25) 399	399
Pharmacist	38 (58)	27 (42) 65	65
Nurse	2432 (54)	2036 (46) 4469	4469
Midwife	369 (71)	146 (29) 515	515

Note: urban areas include Libreville, Port Gentil, Franceville and surroundings.^61^

a Number (percentage).

To fight against diseases, Gabon has developed 17 national health control programs. These programs monitor diseases such as HIV and sexually transmitted diseases, malaria, tuberculosis and also include a wide vaccination program, which covers 100% of the Gabonese territory. Despite the existence of 10 epidemiological stations distributed around the country in both rural and urban areas (Table 2) that act as surveillance outposts, there is no other national health program to combat the more neglected EIDs.

In addition to public health Ministry activities, there are the following in Gabon:

- The CENAREST (National Center for Scientific and Technologic Research) that evaluates and carries out research in Gabon, contributes to the application and promotion of research results and supports research training.
- The CIRMF (International Medical Research Centre of Franceville), a Gabonese world-renowned scientific research center, inaugurated in 1979, the CIRMF had for an initial focus to study the reasons for infertility in Central African populations. By the mid 1980s, CIRMF broadened its research to focus also on tropical diseases including HIV, trypanosomiasis and malaria. More recently CIRMF concentrated also on EIDs including the deadly ebola and marburg viruses, CHIKV and DENV.^63^ Its central position in Africa and its world-renowned researchers ensure that CIRMF benefits from support and collaboration from several international institutions including the WHO (World Health Organization), the CIRAD (Centre de Coopération Internationale en Recherche Agronomique pour le développement), the IRD (Institut de Recherche pour le Développement), the Pasteur Institute, the CDC (Centers for Disease Control and Prevention), and several overseas universities from Europe, North America, South America and Asia.
- International NGOs (non-governmental organizations) are also involved in research on emerging diseases in Gabon. For example, the WCS (Wildlife Conservation Society) with its ‘One World, One Health’ Program and the Zoological Society of London and its ‘Mikongo Conservation Centre’ are working on great ape diseases, wildlife monitoring and the bush meat trade.

#### Regional organizations

In Central Africa there are few regional organizations involved in public health:

- The OCEAC (Organisation de coordination pour la lute contre les endémies en Afrique Centrale) is an organization of coordination and cooperation to fight major endemic diseases in Central Africa. Created in 1963, in Yaounde, by the determination of the health ministers of Cameroon, Congo, Gabon, CAR and Chad, it was originally known as the OCCGEAC until 1965. The Equatorial Guinea joined later the OCEAC. Its goals are to (1) coordinate public health policies and actions in Central African region, (2) participate in the training of the medical staff of member countries of the organization, (3) coordinate applied research projects undertaken by national institutions, (4) implement missions of expertise in the different areas of health sciences, (5) contribute to the public health promotion in the member countries and (6) support the actions undertaken in response to health emergencies. Today OCEAC is in charge of regional health programs and projects like the Sub Regional Program against HIV/AIDS, the Harmonization Program for Pharmaceutical Policy, the Regional Program to fight human African trypanosomiasis and research projects on malaria.
- The CEMAC (Communauté Économique des États d'Afrique Centrale—Economic Community of Central African States) is an economic community of the African Union for promotion of regional economic co-operation in Central Africa. Member countries include Gabon, Republic of Congo, Equatorial Guinea, CAR, Cameroon and Chad. It ‘aims to achieve collective autonomy, raise the standard of living of its populations and maintain economic stability through harmonious cooperation’. It was established in 1983 and its ultimate goal is to establish a Central African Common Market. However CEMAC may have a role in the public health systems in Central Africa: in 2009, CEMAC signed a memorandum of understanding with Germany, which donated a 23 million euro grant for the prevention of HIV in Central Africa.
- The CIESPAC (Centre inter-états d'Enseignement en Santé Publique pour l'Afrique centrale) is a sub-regional public health training institution, originally located in Brazzaville. It was created to provide Central African countries with qualified health service staff and managers. It offers several courses, the most recent of which is recognized with a professional diploma in public health and targets mainly potential health district managers.^64^ The civil war events that occurred in Brazzaville in the late nineties provoked the transfer of the institution to Yaoundé, Cameroon.
- The CAMES (Conseil Africain et Malgache pour l'Enseignement SupérieurFAfrican and Malagasy Council for Higher Education) exists to (1) promote and encourage the understanding and the solidarity between member States, (2) establish a permanent cultural and scientific cooperation between member states, (3) collect and diffuse all academic and research documents, (4) prepare agreement drafts between states concerned by Higher Education and Research and contribute to their implementation and finally (5) develop and promote dialogues to coordinate the higher education system and research so as to standardize programs and recruitment levels. This means that Gabonese university lecturers and researchers (in particular health researcher) are assessed by CAMES before obtaining a promotion.

#### International organizations

Gabon is part of the Global Outbreak Alert and Response Network (GOARN), which contributes towards global health security by,^65^ (1) fighting the international spread of outbreaks, (2) ensuring that appropriate technical assistance rapidly reaches affected countries, and (3) contributing to long-term epidemic preparedness and capacity building. On top of that, the CDC, in conjunction with the WHO, has developed practical, hospital-based guidelines, titled Infection Control for Viral Haemorrhagic Fevers in the African Health Care Setting. This manual helps health-care facilities to recognize cases and prevent further hospital-based disease transmission using locally available materials and only little financial resources (http://www.cdc.gov/ncidod/dvrd/spb/mnpages/dispages/vhf.htm). Moreover, in November 2007, a meeting to discuss the integrated control of neglected zoonotic diseases in Africa was held in Nairobi, Kenya. It was organized by the WHO and jointly supported by the European Commission, the ILRI (International Livestock Research Institute), the Danish Centre for Health Research and Development (formerly the Danish Bilharziasis Laboratory), the FAO (United Nations Food and Agriculture Organization), the OIE (World Organisation for Animal Health) and the African Union.^66^

### Conclusion

Infectious diseases, including zoonoses, remain the major and increasing health threat in most developing countries.^1^, ^3^, ^6^, ^67^ Even if in industrialized countries, cardiovascular diseases and cancers are considered to be the main causes of illness and death, special attention still needs to be paid to zoonotic EID.^67^ This statement is now well described by the ‘one health—one medicine-one world’ concept which is a worldwide strategy for expanding interdisciplinary collaboration and communications in all aspects of health care for humans and animals and the interaction with environmental factors. Also, viral hemorrhagic fevers, because of their high infectiousity and the dramatic outcome, have attracted the attention of the medical world and the public in Africa and around the world to this particular category of EID.^68^

However, global effort in EID surveillance and investigation is inadequately allocated. Indeed, the majority of scientific resources focus on places from where the next important emerging pathogen is least likely to originate.^3^ Jones *et al*. advocated for the re-allocation of resources to EID hotspots in lower latitudes, such as tropical Africa because of the critical need for health monitoring and identification of new potentially zoonotic pathogens in African wildlife populations, and this to be used as a forecast measure for EIDs.^3^, ^48^, ^67^

Like other African countries, Gabonese resources for public health and health monitoring are unequally allocated; 60% are spent at a central level. Public health services and clinical practitioners need more resources to be able to actively educate the public about the risks of repeated contacts with wildlife or other sources potentially harmful for health.^48^ However, Gabon could be considered as a good model to investigate the emergence or re-emergence of zoonotic EID. On one hand, Gabonese forests are a hot spot for biodiversity (wild animals and unknown pathogens) and on the other hand there is a relatively small population (1.5 million of habitants), which is often in contact with surrounding wildlife. Also, the CIRMF, a research center advantageously located, offers high quality researchers and facilities that study pathogens and wildlife ecology. Altogether the combination of these factors should help to better understand the mechanisms of contact and transmission of new pathogens from wildlife to human, the emergence of zoonotic EID and the breaking of species barriers by the pathogens. Indeed the emergence of infectious diseases in wildlife is a continuous and ongoing process. The factors that give rise to zoonotic EID, such as ecosystem perturbations and modifications, climate changes, migrations of reservoirs species, pathogens or vectors, and intrinsic changes of pathogens may be of natural origin or due to human influences.^17^ To understand the underlying mechanisms that govern relationships between reservoir species, ecological factors and environmental perturbations with the emergence, transmission and dissemination of viral diseases in tropical forests, the CIRMF wishes to set up permanent surveillance of the health of the population by the establishment of (1) a network reference laboratories (WHO based reference laboratories including CIRMF, Pasteur Institute Network and other National laboratories or universities based) and (2) a Health Ecology Observatory (that is,. The CIRMF's Scientific Station in la Lopé National Park). Such measures will compile data from the public health system with the monitoring of the emergence of new pathogens. The collected information would favor better outbreak risk appraisal in the Gabonese human population as well as for the entire Congo basin region.
